# Statins as Secondary Preventive Agent to Limit Breast Cancer Metastatic Outgrowth

**DOI:** 10.3390/ijms26031300

**Published:** 2025-02-03

**Authors:** Neha Atale, Alan Wells

**Affiliations:** 1 Department of Pathology, School of Medicine, University of Pittsburgh, Pittsburgh, PA 15213, USA; nea48@pitt.edu; 2 Research and Development Service, Pittsburgh VA Health System, Pittsburgh, PA 15213, USA; 3 Cell Biology Program, Hillman Cancer Center, University of Pittsburgh, Pittsburgh, PA 15213, USA

**Keywords:** anticancer agent, breast cancer, chemotherapy, emergence, extra cellular matrix, immunotherapy, metastatic dormancy, statin, tumor microenvironment

## Abstract

Metastasis is a leading cause of mortality in breast cancer, as metastatic disease is often aggressive and resistant to conventional treatments. Cancer cells that spread to distant organs can enter a dormant phase for extended periods, sometimes years or decades. During this dormant phase, cancer cells avoid immune and pharmacological response. Thus, new approaches are needed to prevent these disseminated cells from becoming lethal cancers. Statins are known inhibitors of 3-hydroxy-3-methylglutaryl coenzyme A (HMG-CoA) reductase that have been extensively used in patients with cardiovascular diseases to lower cholesterol. However, recent research has demonstrated their potential in anticancer therapies. Epidemiological evidence suggests that statins are associated with a reduction in breast cancer-specific mortality, although they do not appear to affect the incidence of primary tumors. In this review, we discuss the role of statins in metastasis and dormancy, their cytocidal and cytostatic effects and their interactions with different cell types in the tumor microenvironment. The exact mechanisms by which statins reduce mortality without influencing primary tumor growth remain unclear, also warranting further investigation into their potential role in metastasis and tumor dormancy, which could ultimately help patients to improve survival and quality of life.

## 1. Introduction

### Breast Cancer Metastasis and Dormancy

Breast cancer is the leading cancer diagnosis among women in the United States, comprising almost 30% of all new cancer cases in women each year. Even with newer targeted and immune therapies, the 5-year survival rate for metastatic breast cancer is only approximately 30%, reflecting the challenges in treating advanced stages of the disease [1]. The American Cancer Society estimates 310,720 new cases of breast cancer to be diagnosed, and about 42,250 deaths are projected in 2024 alone [2,3]. Primary breast cancer is a heterogeneous disease with multiple subtypes, each exhibiting distinct biological behaviors, treatment responses, and prognosis. The most common types include hormone receptor-positive (HR+, estrogen and progesterone receptors), human epidermal growth factor receptor 2 positive (HER2-positive), and triple-negative breast cancer (TNBC), necessitating tailored therapeutic approaches. HR-positive patients, while predominantly cured by local excision and hormonal suppression, still report recurrences in up to 30% with these usually occurring 5 or more years later, with the risk lasting decades [4,5]. HR-negative breast cancers, comprising 20–40% of cases, often lead to distant recurrences within 3–5 years after diagnosis [6]. TNBC is characterized by an aggressive phenotype and accounts for 15% of breast cancers. The 5-year survival rate for patients with metastatic TNBC is lower than for those with HR+, HER2-positive breast cancers [7].

**Figure 1:**
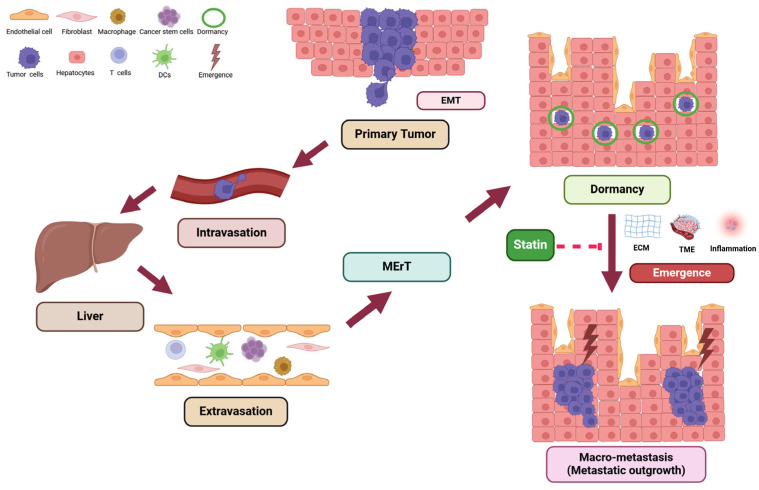
The metastatic progression of breast cancer and role of statin in prevention of metastatic outgrowth. The metastatic process of tumors involves a series of steps, that contribute to cellular dormancy and recurrence during metastasis. Following EMT, tumor cells enter the circulatory system, travel to secondary sites (such as the liver), and exit the blood vessels at these locations. Decades later factors like inflammation, increased stiffness of the surrounding tissue, and the tumor microenvironment of the metastatic organ can trigger the reawakening of these dormant cells, causing tumor recurrence. Statins prevent the reactivation of dormant breast cancer cells at the micro- metastatic site, inhibiting their progression into macro-metastasis (metastatic outgrowth). (Adapted from [32]). This figure was generated using BioRender (version 12-2024) (https://biorender.com, accessed on 21 December 2024).

Thus, the real challenge in breast cancer is its potential for metastasis, where the cancer spreads beyond the breast to other parts of the body, significantly worsening the prognosis. Metastasis refers to the spread of tumor cells from the primary site such as the breasts to other distant locations in the body through a complex, multistep process (Figure 1). Research over the decades has identified how this process occurs in stages: the escape of tumor cells from the primary site, local tissue invasion, intravasation, and finally, exit from the circulation to establish new growths in secondary locations, known as metastatic seeding [8,9]. Disseminated tumor cells lose their epithelial characteristics and acquire mesenchymal traits through a process known as cancer-associated epithelial-to-mesenchymal transition (cEMT), which enhances their mobility and invasiveness. Upon reaching distant sites, these cells often reverse this transition through a mesenchymal-to-epithelial reverting transition (MErT) [10,11,12]. Once they return to an epithelial state, many of these cells can enter a dormant phase that can last for years or even decades. Eventually, these dormant cells may undergo a second epithelial-to-mesenchymal transition (EMT) to induce activation leading to the conversion of micro-metastasis into macro-metastasis (metastatic outgrowth) [13,14].

The multistep process of metastasis begins when cancer cells from primary sites leave and enter the blood circulation in a process called intravasation. These cells in circulation are called circulating tumor cells (CTCs). While many of these cells are eliminated within the bloodstream, some can reach distant organs where they exit the blood (extravasate) and invade organs such as the liver, lungs, brain, and bone marrow. Most of these cells fail to survive in the distant organs, but a small fraction (<1%) enter a quiescent state and can remain inactive for extended periods [15,16,17,18]. In this dormant state, the tumor cells are undetectable and clinically irrelevant. These invading cells are termed as disseminated tumor cells (DTCs). These DTCs can acquire the necessary epigenetic and non-genetic changes to progress to the next stage of metastasis.

The mechanisms that trigger dormant cancer cells to resume growth as aggressive and ultimately lethal metastatic tumors are very critical. Understanding these mechanisms could potentially lead to strategies for preventing further metastases. Several environmental factors and signals can trigger the reactivation of dormant cells. Seminal research in this field has illuminated how signals during tissue inflammation, such as growth factors like epidermal growth factor (EGF), cytokines like Interleukin-8/chemokine (C-X-C motif) ligand 8 (IL-8/CXCL8), and chemokines like CXCL10, can activate dormant cells in ex vivo models. These growth factors and chemokines activate signaling pathways that promote the reawakening of dormant tumor cells, facilitating their transition from a quiescent state to active proliferation and metastasis [19,20,21]. However, this is an active area of research, and a broad discussion of this topic is beyond the scope of this review. We refer readers to these reviews that provide more extensive details [22,23,24]. In general, dormant cells can become active through various external signaling that triggers them into proliferation through various pathways. Gaining further insight into the microenvironmental cues that regulate both dormancy and reactivation is crucial for preventing metastasis and improving long-term outcomes in breast cancer patients.

Current intervention methods to tackle cancer such as chemotherapy and targeted therapies only act on growing or metabolically active cancer cells and are ineffective against dormant cells. Immunotherapies are also ineffective against dormant cells as they downregulate PD-L1 and other targets [25]. Since these dormant cells could become active and outgrow, it is imperative to eliminate them or at least keep them dormant and clinically irrelevant. Recent epidemiological studies have provided correlations between statin usage and reduced cancer metastasis-associated mortality [26,27,28]. As these findings point to a specific reduction in recurrence, without affecting incidence [29,30,31], this points to a repurposing of these well-tolerated agents to limit breast cancer metastatic mortality. In the current review, we aim to summarize the role of statins in influencing tumor dormancy, metastasis, and their overall effect on the tumor microenvironment. While many studies have also considered the effects of statin on primary tumors and their suppression, these are beyond the scope of our review as we focus our discussion on the metastatic aspect of breast cancer and the role of statin in its inhibition as these latter aspects have been reported in clinical correlations in human epidemiologic studies.

## 2. Effect of Statins on Cancer: Mechanisms and Significance

**Figure 2:**
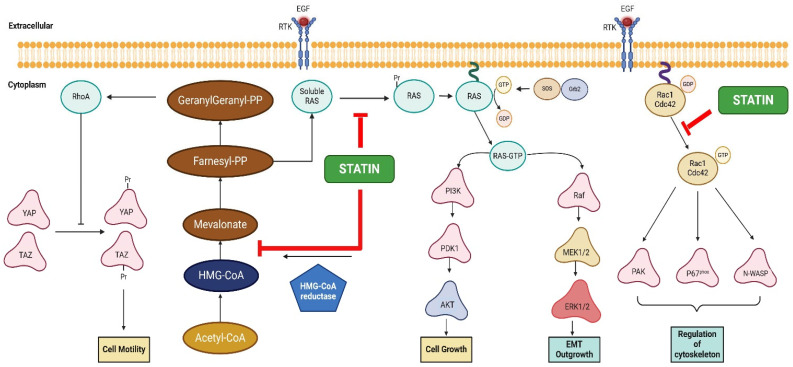
Potential role of statin in inhibition of mevalonate pathway and EMT outgrowth. Statins act on multiple pathways in the cell, it inhibits HMGCR, which facilitates the NADPH-dependent conversion of HMG-CoA into mevalonate. Subsequently, mevalonate is phosphorylated by mevalonate kinase and further processed into IPP. Through the mevalonate pathway, IPP is then used to generate FPP and GGPP, which are essential intermediates in various cellular processes. It blocks the conversion of soluble Ras to the prenylated Ras (Pr-Ras) and thus decreases the prenylation of Ras and other intracellular signaling molecules. Statins localize Ras in the cytoplasm and hence inhibiting AKT activation which eventually reduces tumor cell proliferation. Reduction in ERK signaling by statins inhibits secondary EMT and suppresses outgrowth. Statins also inhibit Rac1/Cdc42 activation and reducing downstream effector activity. Rac1, along with Cdc42, activates a variety of downstream effectors, including PAKs, WASP and p67phox complexes, critical for cell migration and actin polymerization. The inhibition of mevalonate pathway also reduces production of essential downstream end-products such as RhoA which in turn have anti-proliferation effects by decreasing YAP/TAZ transcription, ultimately exerting anti-proliferative effects by inhibiting processes that promote cell growth and division. This figure was created using BioRender (version 12-2024) (https://biorender.com, accessed on 21 December 2024).

Statins are drugs that have been traditionally used to lower cholesterol and reduce the risk of heart attacks [33,34]. They are classified based on their higher affinity to water (hydrophilic) or lipids (lipophilic). Lipophilic statins such as fluvastatin, lovastatin, pitavastatin, simvastatin, and atorvastatin can penetrate cells easily by traversing the cell membrane, whereas hydrophilic statins, rosuvastatin and pravastatin exhibit greater selectivity requiring specific transporters such as OATP1B1 (Organic Anion Transporting Polypeptide 1 B1) and OATP1B3 to access the cytoplasm [35]. Statins lower cholesterol levels through their interaction within the mevalonate pathway, that also leads to decrease in cholesterol precursors which are critical for other signaling pathways. They inhibit 3-hydroxy-3-methylglutaryl-CoA reductase (HMGCR); which plays an important role in cell metabolism and signaling, regulating the production of isopentenyl diphosphate (IPP), farnesyl diphosphate (FPP), and geranylgeranyl diphosphate (GGPP) [36]. Isoprenoid pyrophosphates have attracted significant attention due to their essential role as cofactors in the post-translational modification of key cell-signaling proteins through prenylation. Proteins with a carboxy-terminal CaaX motif are the specific target of this modification. Ras, RhoA, Rac1, Cdc42 are important small GTPases that are essential for cellular signaling and among the proteins which are altered by this process. Many signaling pathways involved in cellular processes such as cytoskeletal rearrangement, cell migration, and division are regulated by these GTPases, which alternate between an inactive GDP-bound state and an active GTP-bound entity. By inhibiting HMG-CoA reductase, statins reduce the availability of these isoprenoid intermediates, thereby affecting the activity of key cell-signaling molecules [37,38]. For example, Ras and Rho proteins are prenylated by farnesyltransferase (FTase) or geranylgeranyltransferase-I (GGTase-I) using FPP or GGPP, respectively. The prenylated Ras proteins, such as K-Ras4A, N-Ras, and H-Ras, are then targeted to the Golgi apparatus, where they undergo further palmitoylation on cysteine residues. This palmitoylation enables the mature protein to traffic to the plasma membrane, where guanine nucleotide exchange factors (GEFs) activate the protein, triggering downstream signaling pathways. Similarly, Rho proteins are chaperoned by a Rho-specific GDP dissociation inhibitor (GDI), which directs them to the appropriate membrane for functioning. Ras proteins primarily regulate cell proliferation and differentiation, while Rho proteins are involved in controlling the actin cytoskeleton, cell migration (critical for cancer cell mitogenesis and tumor progression), gene expression, and proliferation (important in cancer cell mitogenesis and outgrowth). The overall dynamic regulation of these signaling molecules is critical for normal cellular function and their dysregulation is implicated in various diseases, including cancer [39]. Statins localize Ras in the cytoplasm, thereby inhibiting AKT activation and ultimately reducing tumor cell proliferation. Additionally, statins-mediated reduction in ERK signaling impairs secondary EMT and suppresses metastatic outgrowth. Figure 2 depicts the potential role of statins in the inhibition of cholesterol biosynthesis, Ras, Rho, and Rac activation as well as EMT outgrowth.

Statins have been shown to inhibit Ras prenylation and its downstream signaling effectors, such as extracellular signal-regulated kinases 1/2 (ERK1/2), AKT and the mammalian target of rapamycin (mTOR), thereby limiting survival signaling by suppressing GGPP biosynthesis [40,41]. Pitavastatin has demonstrated potent anti-cancer activity in several preclinical cancer models by induction of apoptosis [42]. It triggered the cleavage of caspase-9 and caspase-3 in liver cancer cells. The cytocidal effects were not noted in vivo, pitavastatin rather suppressed tumor growth and improved survival rates in tumor-bearing mice, suggesting a suppressive effect, that is consistent with the epidemiologic correlations in human studies.

Statins exert their effects by inhibiting the mevalonate pathway, which is crucial for the prenylation of Rho GTPases [43,44]. These GTPases require isoprenoid chains, such as the farnesyl or geranylgeranyl groups, for their proper localization and function. Such groups are synthesized from mevalonate and essential for Rho GTPase activation. Once activated, Rho GTPases interact with various intracellular effector proteins to initiate numerous cellular responses, including actin cytoskeleton remodeling, cell cycle progression, apoptosis, adhesion, and gene transcription. Rho GTPases are prenylated through the attachment of FPP or GGPP, catalyzed by FTase and GGTase-I, respectively, as described above. Statins inhibit HMG-CoA reductase, blocking the mevalonate pathway and reducing the production of isoprenoid compounds, which ultimately leads to a decrease in the prenylation of Rho GTPases. Statins can lower the levels of RhoA, a GTPase crucial for maintaining cytoskeletal integrity, which causes cytoskeletal destabilization, which in turn prevents the transcriptional coactivator Yes-associated protein (YAP)from translocating to the nucleus [45]. YAP and its paralog transcriptional coactivator with a PDZ-binding domain (TAZ) are known to drive the expression of genes that promote cancer cell proliferation, migration, and self-renewal of cancer stem cells. By limiting RhoA generation, statins can inhibit YAP/TAZ-driven tumor growth. This is consistent with a suppressive effect on cycling tumor cells.

Tumor dissemination appears to be suppressed by statins in human correlations. Another protein that statins have been shown to regulate is Rac1 which has been implicated in cell motility. Statin has been shown to decrease the phosphorylation of Rac1 in renal cancer cells to prevent angiogenesis, invasion and metastasis [46]. The inhibition of Rac1 by statins can occur through the depletion of these isoprenoids, specifically GGPP, which is necessary for the proper membrane anchoring and activation of Rac1. By inhibiting Rac1, statins can have anti-inflammatory effects and may decrease processes like cell migration and adhesion. Rac1 and Cdc42 activate a range of downstream effectors, such as p21-activated kinases (PAKs), Wiskott-Aldrich Syndrome protein (WASP), mammalian enabled (Mena)/vasodilator-stimulated phosphoprotein (VASP), and p67phox complexes. These effectors are crucial for regulating cell migration and invasion by facilitating actin polymerization and driving cell polarization [47].

In addition to HMG-CoA reductase, histone deacetylase (HDAC) is another target of statins. Statin-induced HDAC inhibition causes acetylated histone H3 to accumulate and p21 expression to rise. This interferes with cyclin/ cyclin-dependent kinase (CDK) complexes, which causes cell cycle arrest and prevents human cancer cells from differentiating. HDACs have been found to be overexpressed in human cancer tissues from a variety of organs, including the stomach, colon, and breast [48]. Most of the research to date has focused on the interaction between HDACs and oncogenic DNA-binding fusion proteins, which facilitates their abnormal recruitment of gene promoters. In the development of acute promyelocytic leukemia and acute myeloid leukemia, for example, fusion proteins like promyelocytic leukemia-retinoic acid receptor alpha (PML-RARα), Promyelocytic leukemia zinc finger-retinoic acid receptor alpha (PLZF-RARα), and acute myeloid leukemia-eight twenty-one (AML1-ETO) recruit HDAC-containing repressor complexes that suppress genes essential for myeloid differentiation [49]. Through the reversal of p21 down-regulation, simvastatin prevented Rho GTPase from being geranylgeranylated, thereby inhibiting the proliferation of mesangial cells induced by high glucose [50]. But lovastatin has also been shown to regulate p21 expression transcriptionally in a way that is independent of p53 [51]. Statins also exhibit anti-cancer properties through several mechanisms, including the inhibition of DNA methyltransferases (DN-MTs), regulation of DNA demethylation, activation of bone morphogenetic protein (BMP) signaling, and promotion of cell cycle arrest via p21 [52]. This results in enhanced growth inhibition of p21 and p27 by preventing their proteasomal degradation. Further research has shown that statins also induce apoptosis in human hematopoietic tumor cells by targeting Ras signaling pathways.

### Anticancer Studies of Statins

By limiting the availability of mevalonate, statins reduce the farnesylation and isoprenylation of small GTPases, leading to a decrease in mitogenic signaling. Most studies suggest that only GGPP can prevent statin-induced apoptosis, supporting the hypothesis that statin treatment depletes cellular GGPP levels. This depletion impairs the isoprenylation of crucial proteins for tumor cell survival and mitogenesis, resulting in their mis-localization and dysfunction, which triggers apoptosis in tumor cells. Furthermore, they increase Bcl-2 interacting mediator of cell death (BIM) expression in human oral squamous carcinoma (HSC-3) and (HEp-2) cells and inhibit Ras membrane localization, which lowers the levels of phosphorylated ERK and mTOR [53].

Prenylated proteins, which typically move from the cytosol to the membrane, play a significant role in signal transduction cascades. Only membrane-associated Ras can interact with membrane receptors to activate downstream signaling pathways; therefore, Ras activation requires farnesylation. It has been suggested that the anti-cancer effects of statins might arise from targeting Ras translocation; however, this theory is debated. Several studies have shown that statins can affect Ras translocation. For example, in mesothelioma cells treated with lovastatin, membrane-associated Ras levels decreased while cytosolic Ras increased. More responsive mesothelioma cell lines displayed greater Ras translocation from the membrane to the cytosol compared to less sensitive lines [54]. In NIH-3T3 cells, transfection with Ras oncogenes enhanced the sensitivity to lovastatin treatment, which also reduced cell invasiveness and Ras membrane localization [55]. Simvastatin, in human smooth muscle cells, prevented the post-translational processing of Ras and inhibited DNA synthesis triggered by Ras/MAPK pathway activation [56]. These studies suggest that Ras farnesylation contributes to statin-induced effects. Our own research also indicated that lipophilic statins, but not hydrophilic ones, could limit AKT signaling in breast cancer cells, reducing cell replication, most likely via limiting Ras signaling.

Geranylgeranylated Rho subfamily proteins were also identified as potential targets, as adding GGPP or geranylgeraniol (GGOH) reversed the effects of statins. For instance, fluvastatin enhanced the cytosolic fraction of RhoA while decreasing its membrane-bound fraction [43]. Similarly, intestinal epithelial cells with Rho inactivated by *Clostridium difficile* toxin B underwent apoptosis and morphological changes as those induced by lovastatin. The amounts of membrane-bound RhoA and RhoB were reduced by lovastatin. The morphological alterations and apoptosis brought on by lovastatin were successfully stopped by cyclo-heximide and geranylgeranylpyrophosphate, but the effect of lovastatin on Rho membrane translocation was not inhibited. By preventing the geranylgeranylation of small GTPases in the Rho family, lovastatin inactivates them, causing morphological alterations and apoptosis [57]. Additionally, it has been observed that atorvastatin inhibits Rho geranylgeranylation, influences the subcellular location and function of Rho proteins, and influences the way melanoma cells spread. In vitro, atorvastatin treatment reversed the metastatic phenotype of human melanoma cells and prevented Rho activation [58]. These findings suggest that geranylgeranylated Rho proteins contribute to the effects of statins in cancer cells.

In non-small cell lung cancer models, atorvastatin was found to suppress Rac1/NADPH oxidase activity and reduction in vascular endothelial growth factor (VEGF) expression and ultimately inhibiting angiogenesis [59]. In cholangiocarcinomas (CCAs), statins induce apoptosis by disrupting the colocalization of Rac with lipid rafts, which reduces Rac1 activity and downregulates ATP-binding cassette transporters such as ABCA1 and ABCG1. These combined effects contribute to the inhibition of CCA cell survival and the disruption of key cellular pathways [60]. These studies suggest the Rac1 signaling could be another important target to prevent metastasis, which suggests that statins can induce Rac1-dependent apoptosis in cells.

Many signaling pathways have been found to be important mediators of the anti-proliferative and pro-apoptotic effects of statins in recent studies [13,61]. Simvastatin, for instance, has been demonstrated to inhibit mTOR activity in glioma cells, downregulate AKT, and activate AMP-activated protein kinase (AMPK) [62]. Human head and neck squamous cell carcinoma cell lines undergo apoptosis when fluvastatin is administered because it increases caspase-3 activation and enhances Bim expression by inhibiting Ras/ERK and Ras/mTOR pathways. In another study, the effects of atorvastatin on the phosphorylation of key signaling molecules, commonly overexpressed in ovarian cancer, such as AKT, ERK, and S6, were investigated. The study showed a significant decrease in S6 phosphorylation in both Hey and SKOV3 ovarian cancer cells. Interestingly, Hey cells exhibited increased phosphorylation of AKT and ERK, whereas SKOV3 cells displayed decreased phosphorylation of ERK [63]. These findings suggest atorvastatin inhibits cell proliferation in various ovarian cancer cells through a variety of mechanisms, as suggested by its varied effects on signaling pathways. While statins exhibit antiproliferative and pro-apoptotic effects in cancer cells, the precise mechanisms may vary depending on the specific cell lines and other factors. However, the exact mechanisms through which statins affect mevalonate-independent pathways remain unclear.

## 3. Statins Exert Anticancer Effects by Promoting Cytocidal or Cytostatic Effects

Cell culture experiments have provided mechanisms for how statins can affect cancer cells directly. These studies point to both cytocidal and cytostatic processes.

Among cytocidal findings, recent studies have shown newer mechanisms of cell death such as ferroptosis, pyroptosis and autophagy. An excess of iron in the cells causes reactive oxygen species (ROS) to build up and lipid peroxidation through the production of peroxide ions, which in turn damages the cells in ferroptosis [64], a type of controlled cell death. A key enzyme in lowering lipid peroxides and preserving cellular redox balance, glutathione peroxidase 4 (GPX4), is closely related to this process. The GPX4-glutathione (GSH)-cysteine axis and pathways like System Xc- contribute to the regulation of ferroptosis by controlling cellular levels of antioxidants and preventing lipid peroxidation. Notably, ferroptosis is closely tied to the mevalonate pathway, which regulates the synthesis of isoprenoids, including isopentenyladenosine (i^6^A). This modification of selenocysteine tRNA is essential for the proper functioning of GPX4. Statins can impair the maturation of sec-tRNA and GPX4 synthesis, thereby depleting i^6^A levels [65]. As a result, statins may sensitize cells to ferroptosis by disrupting antioxidant defenses, specifically by hindering lipid peroxide reduction, making them more vulnerable to oxidative stress and ferroptotic cell death. This link between statins and ferroptosis suggests that statins could potentially be leveraged as a therapeutic strategy to induce ferroptosis in certain cancer cells or other diseases where ferroptosis is desirable.

Pyroptosis [66,67] as the name suggests, involves inflammation within the cells leading to their death. This process is mainly attributed to the activity of the Caspase and Gasdermin protein families. Cell membrane rupture and death can result from either the non-canonical caspase-4/5/11 pathway or the canonical caspase-1 inflammasome pathway. Pyroptosis is a possible mechanism to eliminate tumor cells while not affecting non carcinomatous cells. Simvastatin has been shown to cause pyroptosis in non-small cell lung cancer (NSCLC) in vitro models. Through the upregulation of gasdermin D (GSDMD) and cleaved caspase-1, it triggered pyroptosis and had an inhibitory effect on cell viability [68]. Simvastatin was also found to be effective in reducing colon cancer by triggering pyroptosis via the ROS/caspase-1/GSDMD pathway. The study demonstrated the increase in reactive oxygen species (ROS) activated caspase-1, a key mediator in the pyroptotic process, which in turn cleaved GSDMD. The cleavage of GSDMD resulted in the formation of pores in the cell membrane, leading to cell membrane rupture and the release of pro-inflammatory cytokines. This cascade of events culminated in reduced cell viability in colon cancer cells, highlighting simvastatin’s potential to inhibit cancer cell survival and growth through the induction of pyroptosis [69]. However, further studies need to be performed to explore detailed mechanisms of statins for pyroptosis and whether these effects can be achieved at the doses of statins found in humans.

Autophagy [70], a vital cellular mechanism, plays a key role in maintaining cellular balance by directing proteins, lipids, and organelles to lysosomes for breakdown and recycling. In the context of cancer, autophagy presents a paradox: it can enable tumor cells to endure harsh conditions like hypoxia and nutrient scarcity, while simultaneously acting to limit cellular damage and tumor progression. This dual role makes autophagy a compelling target for cancer therapies. Recent studies revealed that simvastatin could promote autophagy, enhanced cancer cell death through the activation of the ERK1/2 signaling pathway. The combination of pentoxifylline and simvastatin has been shown to have synergistic effects in promoting apoptosis, by a cellular process, autophagy in triple-negative MDA-MB-231 breast cancer cells. Pentoxifylline, known for its anti-inflammatory properties, enhances cellular stress responses, while simvastatin, through the inhibition of the mevalonate pathway, induces oxidative stress [71]. Lovastatin can reduce the proliferation and mobilization of pleural mesothelial cells by triggering autophagy and intervening the Rac/phospholipase C/inositol 1,4,5-triphosphate (IP3) pathway. It inhibits the proliferation and mobilization of pleural mesothelial cells, and induces autophagy, as well as suppressing cell growth and movement. This effect is mediated by the Rac/phospholipase C/IP3 signaling pathway, crucial for cell migration and proliferation and by disrupting this pathway, lovastatin could reduce mesothelial cell expansion, offering a potential therapeutic strategy for diseases with abnormal cell behavior [72]. Autophagy is countered by canonical survival pathways, particularly those involving PI3K and AKT; this is important for statins, as we have published that statins can reduce the level of AKT survival signaling in breast cancer cells [61]. Thus, the question of autophagy is not merely one of statins triggering autophagy, but also of statins decreasing the ability of cancer cells to withstand other triggers of autophagy in the tumor microenvironment. Therefore, strategies to regulate autophagy hold significant potential as therapeutic approaches in cancer treatment.

Lastly, since the earliest events in seeding distant organs include a ‘transient’ dormancy, cytostatic mechanisms would also achieve the goal of rendering these cells clinically irrelevant. The reduction in farnesylation and prenylation of small intermediary signaling molecules would render these cells less responsive to signaling from growth factors and cytokines. Studies conducted in vitro have demonstrated the important role that lovastatin plays in causing cytostatic effects, especially in ACC-MESO-1 human mesothelial cells. These effects are marked by decreased cell migration and reduced cell viability, suggesting that lovastatin impedes the proliferation of cancer cells. Interestingly, rather than triggering apoptosis, the mechanism underlying these effects appears to involve autophagic alterations. As observed in a study by Asakura [72], the function of lovastatin in these cells is not mediated by apoptotic pathways but rather by changes in autophagy, which are crucial for regulating cellular homeostasis. Specifically, lovastatin’s effect was shown to be partially dependent on the Rac/phospholipase C/IP3 signaling axis. This pathway, however, operates independently of mTOR signaling, indicating that lovastatin’s action could involve alternative autophagy-related mechanisms. Further, lovastatin exerts cytostatic effects by upregulating cell cycle inhibitors such as p21WAF1/CIP1 and p27KIP1. These cyclin-dependent kinase inhibitors play pivotal roles in halting cell cycle progression, thus preventing uncontrolled cell division. The upregulation of these inhibitors suggests that statins can modulate cell cycle checkpoints, promote cell cycle arrest and further contributing to the inhibition of cancer cell proliferation [73]. Moreover, simvastatin, been shown to significantly reduce glioma cell proliferation at low concentrations. This reduction in proliferation is mediated by the downregulation of key transcription factors, including activating transcription factor-2 (ATF-2) and c-jun, which are involved in cell survival and proliferation. By inhibiting the transcriptional activity of these factors, simvastatin impedes critical signaling pathways that drive the growth of glioma cells, highlighting the broader potential of statins in targeting various malignancies [74].

Together, these studies underscore the diverse mechanisms through which statin exert their cytostatic effects, particularly their ability to modulate autophagy, the cell cycle, and key transcriptional regulators. While much of the current understanding is based on in vitro models, these findings suggest that statins could serve as promising adjuncts in cancer therapy by targeting multiple cellular pathways essential for tumor growth and progression. However, further studies are needed to fully elucidate the detailed molecular mechanisms of statins and to determine their clinical efficacy in diverse cancer types. Furthermore, the PI3K/AKT signaling pathway has been implicated in resistance to cytostatic drugs, with substantial evidence showing that statins disrupt this pathway across various cancer models. For instance, the MAPK/ERK and PI3K/AKT pathways have been demonstrated to be inhibited by simvastatin in breast cancer cell lines such as MCF-7, MDA-MB-231, and BT-549 [75].

## 4. Preventing Metastatic Outgrowth: The Role of Statins in Cancer Therapy

Statins have emerged as potential agents in cancer therapy due to their correlation with reduced metastatic outgrowth. Some animal model studies have shown direct treatment effects through cytocidal mechanisms [76,77], but these correlations have not been observed in human epidemiological studies or interventional trials [73]. This discrepancy may be due to either the limited concentrations achievable in humans or inherent differences in human cancers, which still need to be determined. However, the cumulative findings are still consistent with using statins in a cytostatic manner to reduce metastatic outgrowth.

Our group has reported that atorvastatin treatment effectively inhibits the outgrowth of dormant breast cancer cells in two spontaneous metastasis models: spleen-to-liver and mammary fat pad-to-lung, as well as in the ex vivo liver micro physiological system (MPS) model [31,32,78]. In all models, atorvastatin successfully limited metastatic outgrowth without affecting primary tumor proliferation; this concurs with the clinical presentation found in epidemiologic studies. However, a study by Tang et al. revealed the reduction in tumor volume by Pitvastatin [79]. They found that the drug reduced cell survival in TNBC cells and the cell death mechanism was attributed to ferroptosis through reduced expression of GPX4 and ferroptosis suppressor protein 1 (FSP1). While the study found the effect of statin on primary tumors, the way it exerted this was through its well acknowledged disruption of the mevalonate pathway. This suggests that depending on the drug and dosage the statin might be able to affect not only the metastatic tumor environments but also primary tumors.

Thus, there appeared to be a selective effect of the statins on the disseminated cells rather than the primary aggressive tumor cells. E-cadherin (E-cad), a protein essential for cell adhesion, is often downregulated in metastatic cancer cells. Atorvastatin was found to suppress the outgrowth of dormant cells with reduced E-cad expression, suggesting its potential to prevent the emergence of quiescent tumor cells and reduce metastatic progression risk. In distinct breast cancer metastasis mouse models, our research evaluated the impact of atorvastatin on metastatic proliferation in the liver in a dose-dependent manner, with similar results observed in a lung metastasis model [31]. This study showed that atorvastatin inhibits PI3K within the PI3K-AKT and MAPK signaling pathways that are crucial in breast cancer cell growth and survival. Statins can significantly reduce the outgrowth of metastatic tumors in breast cancer by modulating key biological pathways involved in cancer cell migration, proliferation, and survival. They exert their anticancer effects through mechanisms such as inhibition of the mevalonate pathway, which reduces the synthesis of essential molecules required for cell growth and motility. This study highlights how statins can disrupt cancer cell signaling, including the Rho family of GTPases, which are involved in cell movement and metastatic spread. Specifically, treatment with atorvastatin dramatically reduced the outgrowth of mesenchymal MDA-MB-231 cells induced by lipopolysaccharide (LPS)/EGF, suggesting that atorvastatin may be an effective way to stop these statin-sensitive cells from emerging from dormancy. This demonstrates its possible function in preventing dormant tumor cells from reactivating.

Interesting findings have been made regarding the reciprocal interactions between disseminated tumor cells and the distinct resident cell populations of the liver, namely parenchymal hepatocytes and non-parenchymal cells (NPCs) like hepatic stellate cells, Kupffer cells, and liver sinusoidal endothelial cells. Because traditional 2-dimensional culture systems and animal models have a number of inherent limitations, it has proven difficult to study the early stages of metastatic seeding, including the crucial choice of whether to enter dormancy or promote outgrowth. Therefore, we have used liver micro-physiological systems (MPS) to mimic the tumor microenvironment in relation to breast cancer metastasis in liver [32]. These MPS are biomimetic multicellular models that include hepatic cells to mimic normal liver functions, perfusion systems to provide oxygenation, nutrients and waste removal to provide long term cultures, and matrices for allowing the cells to organize on and mimic hepatic tissue morphology. This system has the ability to provide readouts from several phenotypic, genomic and other assays.

Specifically, our lab has also developed a model for investigating behavior of cancer cells in hepatic metastatic environment [78]. The Liver Chip is a perusable bioreactor that replicates the structure of the liver sinusoid using a scaffold. Given the importance of species-specific cytokine signaling and metabolism, the Liver Chip stands out from other systems due to its all-human cellular composition, which includes a full complement of donor-matched primary human hepatocytes and NPCs. This apparatus consists of a 12-unit polystyrene platform connected by two chambers: a reactor chamber with high impact polystyrene scaffolds for cell culture and a media reservoir. Within the reactor chamber, resident liver cells (hepatocytes and NPCs) were seeded onto scaffolds and maintained there for up to 15 days with high viability, functionality, and phenotypic retention. Using this model of the hepatic metastatic niche we could study normal liver functions, the role of diurnal fluctuations in the microenvironment, impact of inflammatory states on metastasis and study the crosstalk between hepatic and tumor cells in the microenvironment.

Others have found similar effects of the statins on metastasis in experimental models. Simvastatin pretreatment has demonstrated an impact on distant brain metastasis in breast cancer mouse models. Mice injected with SUM 149 breast cancer cells, either orthotopically or via the tail vein, exhibited reduced brain metastasis and extended metastasis-free survival in the simvastatin-treated group compared to the dimethyl sulfoxide (DMSO) control group. In these models, Simvastatin down regulated phosphorylated Forkhead box O3 (FOXO3a) and AKT. In the presence of EGF, simvastatin prevented FOXO3a from becoming phosphorylated and preserved the amount of total FOXO3a [80]. Additionally, bone marrow, a common site for breast cancer metastasis, showed fewer osteolytic lesions in simvastatin-treated mice, indicating its potential to prevent bone metastasis. Here statins were found to decrease the expression of CD44 through reduction of CD44 mRNA in both MDA-MB-231 and BT-20 cells indicating the role of statins in limiting CD44 through transcriptional mechanisms [81]. Furthermore, three weeks of fluvastatin treatment alone and in combination with zoledronic acid significantly lowered the overall metastatic burden in a xenograft mouse model. Transcriptome analysis was performed to examine the gene expression changes induced by both treatments, fluvastatin and zoledronic acid which significantly inhibited the metastasis of breast cancer cells, but through different mechanisms [82]. Given the global and organ-specific benefits observed in these metastatic breast cancer models, further research is warranted to explore the clinical relevance of statins’ anti-metastatic properties in prospective clinical trials.

## 5. Tumor Microenvironment in Metastasis: From Cancer Progression to Metastatic Outgrowth

The above discussions posit a direct effect of the statins on the disseminated cancer cells. However, as these cells are phenotypically plastic based on their nature and change their behavior in response to their microenvironment [13,18,31,61,83,84,85,86], it must be considered that statins may affect metastatic outgrowth by modulating the tumor microenvironment at the ectopic sites.

**Figure 3:**
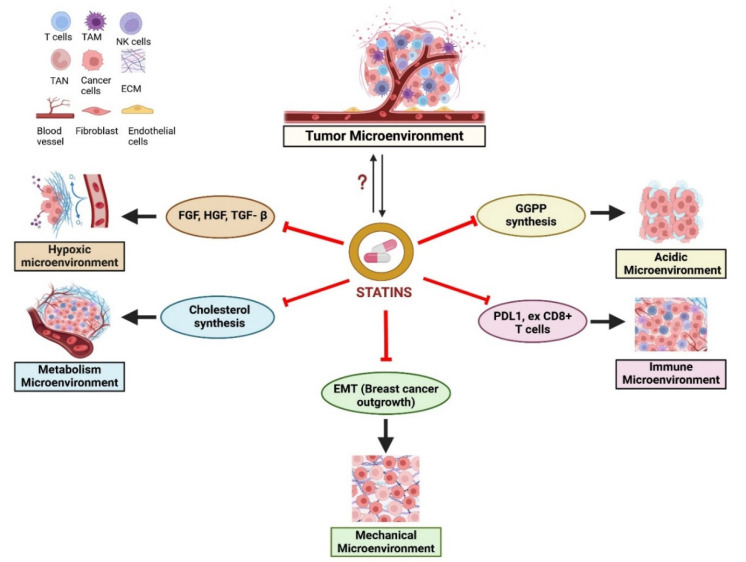
Potential targets of statins in tumor microenvironment. Statins may influence key features of the TME, including hypoxia, acidity, metabolic processes, and immune response. However, the precise role of statins in modulating the TME requires further exploration. The interaction between cholesterol metabolism and TME is integral to tumor development and cancer progression. Statins could alter the TME by disrupting Ras/ERK and Ras/AKT signaling pathways, leading to reduced levels of growth factors (FGF, HGF, etc.) and a decrease in exhausted CD8+ T cells. (This figure was created using BioRender (version 12-2024) (https://biorender.com, accessed on 21 December 2024). GGPP: geranylgeranyl diphosphate, PDL1: Programmed Death Ligand-1, FGF: Fibroblast Growth Factor, HGF: Hepatocyte Growth Factor, TGFβ: Transforming Growth Factor β, TAM: Tumor-associated macrophages, ECM: Extracellular matrix, TAN: Tumor-associated neutrophils.

The tumor microenvironment (TME) in breast cancer metastasis is crucial in promoting tumor development and treatment outcomes, making it essential for effective disease management. It is considered that TME is not merely a passive backdrop; however, it actively drives cancer progression. The awakening and proliferation of cancer cells is highly affected by their microenvironment which also dictates the effectiveness of any potential therapeutics. Cancer evades the defense mechanisms and reprogram cells within the TME to support tumor growth. This allows the cancer cells to proliferate, invade out of their primary site and intravasate into the blood stream. At distant locations, the TME can dictate the conditions for extravasation and probabilities of cancer cell survival. During metastasis, the TME plays a pivotal role in regulating metastatic cell dormancy, their reactivation, and subsequent expansion. TME being the natural cell environment is constituted by different cell types such as immune cells, stromal cells, fibroblasts, endothelial cells and the extracellular matrix, which interact with cancer cells to promote tumor growth [17,87,88] and play a crucial role in influencing metastasis and the potential for recurrence [89]. Our group has identified potential biomarkers for actively proliferating and dormant metastatic cells, which highlight the distinct signals associated with each cell state [90]. It is comprised of various aspects, including the hypoxic, immune, metabolic, acidic and mechanical niche. The emerging cancer cells can change the TME to enhance their chances of survival and facilitate future metastasis and invasion. To counteract the challenges of hypoxia and acidity, the TME initiates processes that promote angiogenesis, ensuring a renewed supply of oxygen and nutrients while clearing metabolic waste. Figure 3 highlights the potential targets of statins in the tumor microenvironment, illustrating how statins may modulate various cellular and molecular components that contribute to metastasis progression.

Tumors are also infiltrated by a variety of immune cells, which can exert both tumor-promoting and tumor-suppressing effects. Emerging evidence marks the potential of immune and stromal gene signatures as valuable tools for predicting prognosis and treatment response in breast cancer. Statins modify the TME by impacting the non-cancerous cells and their associated components within the tumor, including the molecules they secrete and produce. The key components of TME along with their role in tumor development, are mentioned below, as a prelude to the section following that addresses how these aspects are impacted by statins.

### 5.1. Tumor Associated Macrophages (TAMs)

Tumor-associated macrophage (TAM) accumulation in breast cancer is driven by resident macrophages and the recruitment of circulating monocytes. Traditionally, TAMs are classified into M1-like and M2-like macrophages. M1-like macrophages, activated through classical pathways involving interferon-γ (IFN-γ) combined with lipopolysaccharide (LPS), are known for their anti-tumor properties. In contrast, M2-like macrophages, activated by IL-4, and are associated with tumor-promoting activities. Immunosuppressive or pro-angiogenic traits have also been identified within the TAM population in the TME [91]. Generally, the presence of M1 macrophages in the TME reduces tumor aggression, whereas a predominance of M2 macrophages is linked to accelerated tumor growth and a worse prognosis. Our group has also investigated the significant impact of macrophage polarization, specifically the M1 and M2 subtypes on regulating EMT. M2 macrophages promote metastatic outgrowth, while M1 macrophages could play a role in maintaining dormancy in metastatic breast cancer cells. Consequently, the EMT and MErT processes appear to be inhibited by the macrophage phenotype present in the liver metastatic microenvironment. These findings highlight macrophages as a potential therapeutic target for limiting mortality associated with malignant metastases in breast cancer [92]. The liver’s unique immune landscape provides a complex backdrop for macrophage-tumor interactions, with M1 and M2 macrophages potentially shaping the fate of metastatic breast cancer cells in this niche. Simvastatin has been shown to re-polarize TAMs, promoting a shift from the M2- like to M1- like phenotype and inhibiting EMT in lung cancer through cholesterol-related liver X receptor (LXR)/ABCA1 regulation. This led to an increased production of TNF-α and a reduction in TGF-β levels, which in turn remodeled the TME and inhibited EMT process [93]. Statins also affect macrophages in other ways that are advantageous not only in cancer but in many other diseases [94]. For example, several pravastatin, atorvastatin, and simvastatin treatments have been demonstrated to lower the M1/M2 ratio and encourage polarization from the M1 to M2 phenotype for anti-inflammatory effects [95]. Additionally, statins have been shown to suppress the expression of the CD47 molecule, which increases macrophages’ phagocytic capacity. Statins also improve programmed cell removal by preventing NF-κB1 p50 from nuclear translocating and lowering the expression of CD47, the crucial ‘don’t-eat-me’ signal. The phagocytic capacity of macrophages is increased by this action, which also reinforces the additive anti-atherosclerotic effects of CD47–Inhibitory signal regulatory protein α (SIRPα) blockade [96]. Given their involvement in regulating both tumor dormancy and outgrowth, targeting macrophage polarization or specific macrophage-driven signaling pathways may offer a novel therapeutic approach to limit metastatic spread and reduce mortality associated with advanced breast cancer.

### 5.2. T Cells

Statins may amplify T cell activity by enhancing CD8+ T cell-mediated cancer cell killing through the perforin/granzyme or FASL-FAS pathway. They have also been shown to reduce T cell exhaustion within the tumor microenvironment, potentially improving the effectiveness of immune responses. Additionally, statins may modulate CD4+ T cell function, supporting Th1-mediated antitumor immunity while limiting Th2-induced tumor progression. Regulatory T cells (Tregs) are an important part of the TME by modulating immune responses and maintaining homeostasis. These cells are often induced and differentiated from conventional T cells within the TME and exhibit potent immunosuppressive functions. They are known to suppress the activity of immune effector cells through various mechanisms, making them critical players in tumor immune evasion [97,98]. Janghorban et al. identified a few small aggregates of T cells (CD4+ and CD8+) during the dormant stage. The study observed an increased expression of Th2, Lag3, Cd244a, and Pdcd-1 in the primary tumor. In contrast, during dormancy, there was an increase in Th1 cell activity, alongside a decrease in Tregs and Th2 cells [99]. By reducing Tregs, statins could help reduce immune suppression, potentially improving the efficacy of immune checkpoint blockade therapies.

Beyond cancer, statins also influence T cells in various conditions, particularly by enhancing their anti-inflammatory effects. For example, simvastatin has been shown to improve the survival rate of patients with *Staphylococcal bacteremia* by inhibiting superantigen-mediated T cell activation. Superantigens, which are produced by certain bacterial toxins, can overstimulate T cells, leading to excessive immune responses, severe inflammation, and tissue damage. Additionally, atorvastatin has been found to inhibit the expression of histocompatibility class II antigens, further modulating immune responses and potentially reducing inflammatory damage in a range of immune-mediated conditions [100]. In another study, simvastatin reduced low-density lipoprotein (LDL) cholesterol levels and inhibited T cell activation by preventing prenylation, which contribute to an inhibition of atherosclerosis [101].

### 5.3. Cancer-Associated Fibroblasts (CAFs)

Cancer-associated fibroblasts (CAFs) are key stromal components in the tumor microenvironment, particularly in metastasis. These cells are classified into distinct subtypes, including myofibroblasts, metastasis-associated, inflammatory, and antigen-presenting fibroblasts, each with unique characteristics and transcriptomic plasticity [102,103]. A hallmark of CAFs is their ability to synthesize and remodel the extracellular matrix (ECM), interact with immune cells, and support cancer cell proliferation and angiogenesis. Additionally, CAFs play a crucial role in promoting inflammation and fibrosis, which contributes to tissue stiffness-a factor that has been linked to poorer survival outcomes in pancreatic and breast cancer patients [104]. Statins, by inhibiting TGF-β production, may prevent the differentiation of fibroblasts into the pro-fibrotic myofibroblast subtype, potentially reducing fibrosis and creating a less aggressive tumor microenvironment.

Atorvastatin has been reported to reduce fibrosis in cardiac fibroblasts by preventing the formation of advanced glycation end products (AGEs) through the activation of PPAR-γ. This leads to a decrease in RAGE expression, which in turn alleviates the inflammatory and profibrotic effects triggered by AGEs. Additionally, atorvastatin inhibits the phosphorylation of ERK1/2, a key signaling pathway activated by AGEs that plays a critical role in fibroblast proliferation and the progression of fibrosis. Through these mechanisms, atorvastatin helps mitigate the fibrotic processes that contribute to cardiac tissue remodeling [105]. Atorvastatin treatment led to improved graft take and accelerated wound closure in full-thickness wounds, with a faster resolution of neutrophils compared to all other treatments. Additionally, atorvastatin reduced the presence of alpha-smooth muscle actin-positive cells, indicating a decrease in fibrosis, when compared to the control treatment. These provide evidence of a wide range of anti-fibrosis effects of statins [106].

### 5.4. Endothelial Cells (ECs)

The endothelium of the blood vessels is a thin, heterogeneous layer of highly specialized endothelial cells (ECs) that line the inner surface of blood and lymphatic vessels. Tumor-associated endothelial cells exhibit distinct abnormalities in their phenotype, gene expression patterns, and functional characteristics. Tumor growth leads to the development of a hypoxic microenvironment that, in conjunction with oncogenic processes, triggers angiogenesis [107]. Intravasation is initiated when tumor cells attach to endothelial cells. The new blood vessels formed through angiogenesis surrounding the tumor do not have fully formed extra-cellular structures that facilitate the extravasation of the cancer cells. The colonization of circulating tumor cells (CTCs) in the liver results in the development of liver metastasis. Additionally, liver sinusoidal endothelial cell (LSEC) capillarization plays a critical role in the establishment of metastatic lesions in the liver. In models of non-alcoholic steatohepatitis (NASH), LSECs have been shown to undergo dedifferentiation, contributing to alterations in the hepatic vasculature and promoting disease progression. Notably, statin treatment has been found to restore the healthy phenotype of LSECs, reversing their dedifferentiation and improving vascular function [108]. Simvastatin has been shown to inhibit the GGTase-RhoA-YAP-SRY box transcription factor 9 (SOX9) signaling pathway, thereby improving endothelial-to-mesenchymal transition (EndMT)-associated endothelial functions through its interaction with YAP-related signaling. In diabetic conditions, simvastatin significantly enhances the function of human induced pluripotent stem cell-derived endothelial cells by reducing chromatin accessibility at transcriptional enhancer-associated domain elements. This reduction alters the expression of genes involved in EndMT, with the effect being mediated through a YAP-dependent mechanism. SOX9, positioned downstream of statin–YAP signaling, plays a pivotal role in promoting EndMT. Ultimately, these actions contribute to the restoration of endothelial cell function, particularly under diabetic conditions [109]. Based on these findings, it is hypothesized that statins may similarly promote the function of endothelial cells within the TME, potentially restoring their normal phenotype and impacting tumor vasculature. However, there is no direct data currently existing to support this hypothesis, and further investigation is needed to elucidate the potential therapeutic effects of statins on endothelial cell behavior in the TME undergoing metastasis.

### 5.5. Extracellular Matrix (ECM)

In the TME, the extracellular matrix (ECM) is predominantly produced by CAFs, secreting proteins at levels significantly higher than normal fibroblasts. Cancer cells interact with the ECM via TME through receptors such as CD44 and integrins, which are crucial part of the signaling pathways involved in tumor progression [110]. The interstitial matrix, primarily composed of stromal cells, is rich in fibrillar collagens and proteoglycans. Cancer cells, CAFs, and TAMs driven by hypoxia, collectively alter the ECM within the TME by increasing the accumulation of biophysical components such as collagens and enhancing cross-linking through enzymes from the Lysyl Oxidase (LOX) family, including LOX-1, LOXL-2, and transglutaminase enzymes like transglutaminase-2 [111,112]. For instance, a stiffer ECM leads to integrin clustering, which activates FAK/Src complexes and triggers downstream ERK and PI3K/AKT signaling, supporting cancer cell proliferation and survival [113,114]. Previous studies have reported that Simvastatin degrades ECM formation and reduces collagen expression [115,116]. A study demonstrated that the administration of atorvastatin in diabetic neuropathy led to a reduction in the levels of extracellular matrix (ECM) molecules, including collagen IV and fibronectin. This effect was linked to atorvastatin’s ability to inhibit the increased secretion of platelet-activating factor (PAF) and the activation of the PKC-TGF-β1 signaling pathway, both of which contribute to the pathogenesis of diabetic neuropathy [117]. In cardiovascular disease, statin was found to regulate lox expression that could be disrupted by RhoA/Rho kinase-based mechanisms [118]. Due to these possible mechanisms, a deeper understanding of how statins impact ECM dynamics could reveal new therapeutic strategies to prevent or reduce metastatic spread in cancers, particularly in the context of breast cancer-related malignancies.

## 6. Conclusions and Future Perspectives

Statins have shown a broad spectrum of anti-cancer effects in different breast cancer models, offering insights that span the continuum of cancer treatment. They are generally well-tolerated and have a favorable safety profile, making them attractive candidates for adjuvant cancer therapy. The varying effectiveness of statins on primary versus metastatic tumor cells warrants further investigation. While statins play a significant role in modulating the transition between epithelial-like and mesenchymal-like states in cancer cells, potentially impeding the metastatic process, it is also possible that in 3D culture models, a higher density of mesenchymal cells produces autocrine factors that can overcome the growth inhibition caused by limited prenylation, a key effect of statins. They specifically block mesenchymal-epithelial reversal transition or inhibit the proliferation of reverted mesenchymal cells remains an open question.

Clinical trials have demonstrated the well-established role of statins in reducing mortality and recurrence in breast cancer patients. However, despite these promising outcomes, statins do not appear to inhibit the incidence of primary cancer itself. This observation underscores the need for further research into how statins interact with both primary and metastatic tumor environments, and how they might influence already established therapeutic mechanisms in breast cancer treatment. One prospect is that statins could block tumor cell proliferation, which may inadvertently interfere with therapies that rely on the activation of cell cycle for effectiveness. For instance, many chemotherapies and targeted therapies, such as those that inhibit HER-2 signaling, are most effective when tumor cells are actively dividing. Therefore, understanding how statins alter cell cycle dynamics in this context, is essential to optimize their role as adjuvant therapy.

Another important consideration is the role of statins in the metastatic cascade. The ability of statins to modulate the tumor microenvironment—by influencing immune cell recruitment, angiogenesis, or extracellular matrix remodeling—could significantly affect the spread of cancer to distant organs. The effects of statins in many different cell types in metastatic tumor microenvironment remain unexplored. By delving deeper into the mechanisms through which statins prevent metastatic recurrence, we can tailor their use in clinical settings, developing personalized treatment plans that maximize their therapeutic benefit. This is especially crucial for high-risk breast cancer subtypes, such as HER-2 positive or TNBCs, where the probability of metastasis or relapse is particularly high. For patients with these aggressive forms of cancer, statin therapy could offer substantial benefits, potentially improving long-term survival outcomes by preventing metastatic spread or recurrence after initial treatment.

In the future, understanding the mechanisms through which statins impact tumor progression, and metastasis will be crucial for optimizing their therapeutic use in breast cancer. This research could lead to more effective and personalized treatment strategies, offering significant benefits to patients, particularly those at high risk of metastatic relapse. Incorporating statins into cancer treatment regimens could significantly enhance survival rates and improve the quality of life for cancer patients. Harnessing statins—a widely accessible and well-established therapy proven effective in treating cardiovascular disease—presents a promising, cost-effective strategy to transform cancer care, offering hope for improved patient outcomes.
